# Tollip Is a Mediator of Protein Sumoylation

**DOI:** 10.1371/journal.pone.0004404

**Published:** 2009-02-09

**Authors:** Alessia Ciarrocchi, Romina D'Angelo, Chiara Cordiglieri, Ada Rispoli, Spartaco Santi, Massimo Riccio, Simona Carone, Anna Laura Mancia, Simone Paci, Elena Cipollini, Davide Ambrosetti, Marialuisa Melli

**Affiliations:** 1 Department of Biology, Bologna University, Bologna, Italy; 2 Institute of Organ Transplantation and Immunocitology-ITOI, C.N.R, Bologna, Italy; Johns Hopkins University, United States of America

## Abstract

Tollip is an interactor of the interleukin-1 receptor involved in its activation. The endosomal turnover of ubiquitylated IL-1RI is also controlled by Tollip. Furthermore, together with Tom1, Tollip has a general role in endosomal protein traffic. This work shows that Tollip is involved in the sumoylation process. Using the yeast two-hybrid technique, we have isolated new Tollip partners including two sumoylation enzymes, SUMO-1 and the transcriptional repressor Daxx. The interactions were confirmed by GST-pull down experiments and immunoprecipitation of the co-expressed recombinants. More specifically, we show that the TIR domain of the cytoplasmic region of IL-1RI is a sumoylation target of Tollip. The sumoylated and unsumoylated RanGAP-1 protein also interacts with Tollip, suggesting a possible role in RanGAP-1 modification and nuclear-cytoplasmic protein translocation. In fact, Tollip is found in the nuclear bodies of SAOS-2/IL-1RI cells where it colocalizes with SUMO-1 and the Daxx repressor. We conclude that Tollip is involved in the control of both nuclear and cytoplasmic protein traffic, through two different and often contrasting processes: ubiquitylation and sumoylation.

## Introduction

Tollip is an interactor of the Toll family of proteins, which are highly conserved in evolution from *Drosophila* to *Man*, and include the Toll-like and interleukin-1 (IL-1) receptors, all involved in the inflammatory response. The sequence of Tollip, spanning 274 amino acids, contains some characterized functional domains. A C2-like domain, lacking the two conserved Asp residues critical for Ca^2+^ binding and present in numerous eukaryotic proteins as a binding site for a variety of phospholipids [1]. A C-terminus ubiquitin binding CUE domain identified by Shih et al. [2] as a ubiquitin binding site that mediates intramolecular monoubiquitylation. At the amino-terminus (aa 1–54), a GAT/TOM1 interacting domain is also described [3]. So far, Tollip has been involved in two main functions. The first, proposed by Burns and collaborators [1], indicates Tollip as an interactor of the IL-1 receptor TIR domain, mediating the binding of the serine/threonine kinase IRAK-1 to the activated receptor complex, thus being integral part of the IL-1RI signaling cascade. Later, Yamakami & Yokosawa [4] proposed a negative regulatory role of Tollip on the IL-1β and TNF-α signaling pathways, in agreement with the inhibition of NF-kB activation observed following Tollip overexpression [1], [5]. The second function, described by Yamakami et al. [3], concerns the interaction of Tollip with Tom1, Ubiquitin and Clathrin in a high molecular mass complex involved in protein sorting. In agreement with this proposal, an endosomal function of the protein was suggested by Katoh et al., [6], [7]. In addition, Brissoni et al. [8] have recently shown that Tollip is required in the sorting of the IL-1RI at late endosomes, further clarifying its involvement in the IL-1 inflammatory pathway. Zhang and Ghosh [5] have shown that Tollip associates not only with IL-1RI but also with the TLR2 and TLR4 receptors when activated by LPS stimulation. Also this interaction results in the suppression of TLR-mediated cellular responses through the inhibition of phosphorylation and kinase activity of IRAK1.

We show, for the first time, the involvement of Tollip in the sumoylation process. Sumoylation is a post-translational modification of proteins that conjugate SUMO-1 with a lysine of the target peptide through the formation of a covalent bond. This reaction is very similar to the ubiquitylation process and it is catalyzed by three enzymes, that act in succession: 1) El, the dimeric activation complex, composed of the proteins Aosl and Uba2; 2) the single E2 conjugation enzyme named Ubc9; 3) at least three types of E3 ligases. The first group of E3 ligases is encoded by the protein inhibitor of activated signal transducer and activator of transcription (PIAS) family and entails the 5 mammalian members: PIAS1, PIAS3, PIASxα/ARIP3 , PIASxβ, and PIASy. These ligases are characterized by the presence of a RING-finger-like structure essential to their function. The second group is represented by the RanBP2/Nup358 protein, which is a component of the nucleopore complex and does not contain the ring finger domain [9]. The third type is Pc2, a member of the polycomb group of proteins (PcG) involved in gene silencing. Pc2 recruits the CtBP repressor to the PcG nuclear bodies and enhances its sumoylation [10]. These ligases are not protein specific and show overlapping targets. In fact, it is not clear how the specificity of the sumoylation reaction is achieved. It seems likely that more ligases or cofactors exist and contribute to the specificity of the modification process.

In order to further characterize the function of Tollip, we have screened a cDNA library by the yeast two-hybrid system, looking for Tollip partners. In addition to the known interactors, TOM1, Tollip itself and ubiquitin, we have isolated Daxx, SUMO-1, and the E2 (Ubc9) and E3 (ARIP3) sumoylation enzymes. This work shows that Tollip binds Ubc9, SUMO-1 and at least three ligases of the Pias family and is itself sumoylated. The binding domain of Tollip to the ligases of the PIAS family and of the ARIP3 ligase to Tollip are identified. In particular, in a SAOS-2 clone enriched in IL-1RI receptors (SAOS-2/IL1R), Tollip translocates into the nucleus where it co-localizes with SUMO-1 and Daxx, in nuclear bodies. The SAOS-2/IL1R clone was derived from the SAOS-2 osteoblastoma cell line which constitutively expresses IL-1RI [11], [12] and was kindly provided by Dr Sandra Marmiroli (Modena and Reggio Emilia University). Our results may correlate with the interaction of Tollip with the RanGAP-1 protein. Furthermore, we identify the cytoplasmic domain of IL-1RI as a target of Tollip sumoylation. We propose that Tollip is involved in the nuclear translocation of proteins either as a sumoylation cofactor or a ligase.

## Results

### Tollip interacting proteins

**Figure 1:**
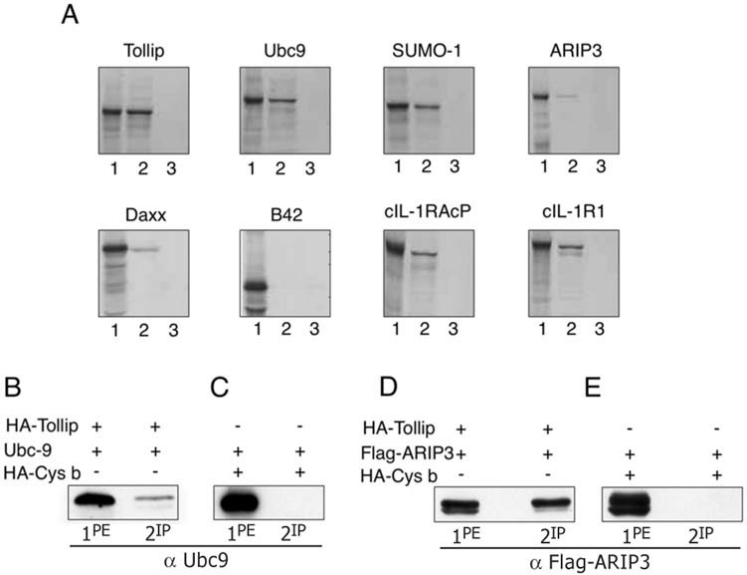
Tollip interactions. A) GST-Tollip interaction with ^35^S-methionine labelled proteins isolated by the yeast two hybrid technique. Lane 1 contains 1/10 input of the ^35^S-methionine protein used for each interaction; lanes 2 and 3 contain the elution product from incubation of the ^35^S-proteins with the GST-Tollip and GST-protein alone, respectively.^ 35^S-methionine labelled proteins were visualized by autoradiography. Western blot analysis of 293T cells transfected with Ubc9 and HA-Tollip (B), Flag-ARIP3 and HA-Tollip (D), HA-Cystatin B and Ubc9 (C), HA-Cystatin B and Flag-ARIP3 (E). HA-Cystatin B is a ubiquitous protein with antiprotease function, unrelated to Tollip, nor to the inflammatory pathway [28]. Lane 1 contains the protein extract; lane 2 contains the proteins immunoprecipitated with anti-HA abs. In this and in the following figures, “pe.” refers to the protein extract and “ip.” to the immunoprecipitated protein. Staining carried out as indicated under the figures.

The yeast two-hybrid mating assay was carried out as described in material and methods. High stringency screening of a cDNA library from rat brain hemispheres, allowed the isolation of 310 positive recombinant clones that were analyzed by PCR amplification. 213 cDNA recombinants showed a single band of amplification that was further characterized. Table 1 lists some of the isolated cDNAs (column 1) with the corresponding aminoacid regions (column 2) and the number of independent colonies for each isolated recombinant clone (column 3). The majority of cDNA recombinants were coding for ubiquitin or ubiquitin fused to ribosomal RNA subunits. This is in agreement with the presence of a ubiquitin binding CUE domain at the C-terminus of Tollip and with the described cytoplasmic role of ubiquitinated Tollip [8], [2], [3]. The already described binding of Tollip to TOM1 and its homodimerization are consistent with the published results [3], [5]. Interestingly, proteins involved in the sumoylation process (SUMO-1, Ubc9, ARIP3) and the transcriptional repressor Daxx are among the interactors that we have isolated. The interactions of Tollip with some of the identified proteins were confirmed by GST-pull down experiments. The B42 fused ^35^S-peptides were incubated with GST-Tollip or GST beads and the interaction was analyzed by SDS-PAGE and authoradiography (Fig. 1A). All the peptides analyzed interact with Tollip but not with the GST control. B42 alone does not interact with Tollip, showing the specificity of the reaction. The cytoplasmic region of the IL-1RI accessory protein (IL-1RAcP) and IL-1RI were used as positive control. To confirm the interactions observed in yeast, 293T cells were transfected with the pRK7-HA-Tollip cDNA together with constructs coding for Ubc9 (Fig. 1B ) or the Flag-ARIP3 fusion proteins (Fig. 1D). 293T cells expressing the unrelated protein HA-Cystatin B, together with Ubc9 (Fig. 1C) and Flag-ARIP3 (Fig. 1E) were used as control. The immunoprecipitation with anti-HA antibodies (abs) shows that Ubc9 (B) and Flag-ARIP3 (D) co-immunoprecipitate with HA-Tollip but not with the control protein (C and E), confirming the specificity of the binding.

**Table 1:** 

cDNA	Isolated protein fragment	N° of independent colonies
**Ubiquitin conjugating enzyme 9 (Ubc9)**	1–159 aa (full length)	45
**Death domain interacting protein (Daxx)**	643–739	5
**Target of Myb-1 homologue (TOM1)**	3–492	**5**
**Androgen receptor interacting protein 3 (ARIP3)**	64–572	3
	448–572	2
**Small ubiquitin-like modifier 1 (SUMO-1)**	1–101 aa (full length)	1
**Tollip**	141–274	1
**Leucin Rich Protein**	671–832	1
**Brain expressed X-linked 2 (Bex1)**	33–129	1
**Receptor for Activated C Kinase (RACK 1)**	241–317	1
**ING 1 tumor suppressor**	248–276	1
**Ubiquitin family**		
**Ubiquitin**	1–76 (full length)	71
**Ubiquitin/A52 fusion protein**	72–105	7
**Ubiquitin/S27 fusion protein**	2–157	4

### Tollip interaction with the PIAS family of proteins

**Figure 2:**
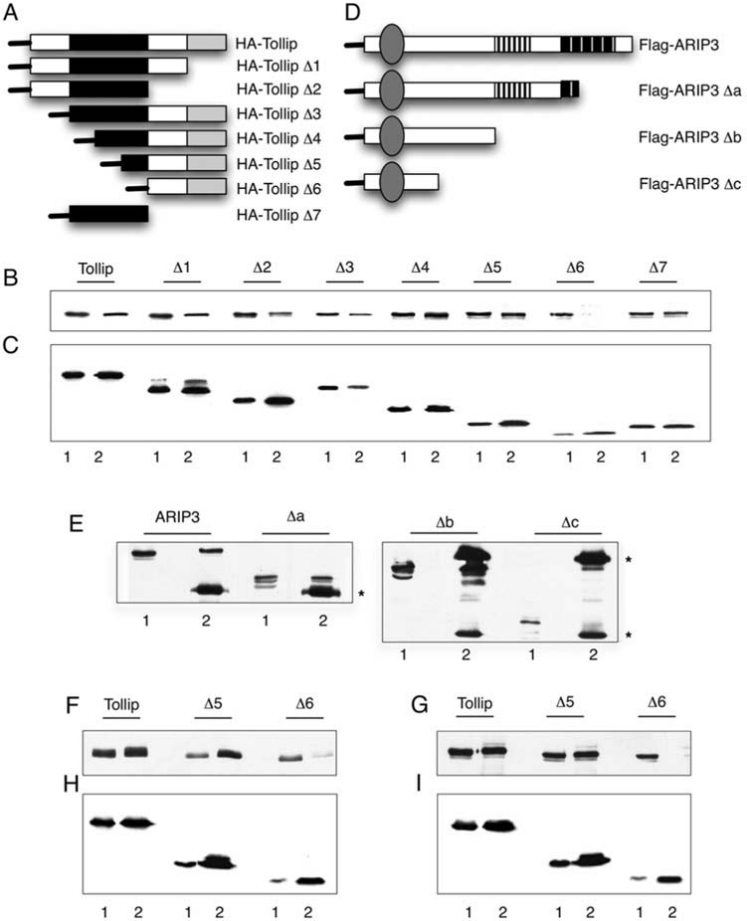
Tollip and ARIP3 interacting domains. A) Schematic representation of HA-Tollip deletion mutants. The black box represents the C2 domain (aa 54–179), the grey box represents the CUE domain (aa 229–274). HA-Tollip wt: aa 1–274; Δ1: aa 1–229; Δ2; aa 1–179: Δ3: aa 54–274; Δ4: aa 94–274; Δ5: aa 134–274; Δ6: aa 179–274; Δ7: aa 54–179. In all western blots described in this figure, Lane 1 contains the protein extract and lane 2 the immunoprecipitated proteins. B) Western blot of protein extracts from 293T cells, transfected with Flag-ARIP3 cDNA and with the indicated HA-Tollip deletion mutants, before (lane 1) and after (lane 2) immunoprecipitation with anti-HA abs. The staining is with anti-Flag abs. C) Western blot from the same protein extracts as in B stained with anti-HA abs. D) Schematic representation of the Flag-ARIP3 deletion mutants. The circle represents the SAP domain (aa 11–45), the white box with black stripes represents the RING domain (aa 347–388) and the black box with white stripes represents the AR-ID domain (aa 443–548). Flag-ARIP3 wt: aa 1–572; Δa: aa 1–467; Δb: aa 1–347; Δc: aa 1–169. E) Western blot of protein extracts from 293T cells transfected with HA-Tollip and the indicated Flag-ARIP3 deletion mutants. The protein extract was immunoprecipitated with anti-HA abs and stained with anti-Flag antibodies. F) Western blot of protein extracts from 293T cells transfected with the Flag-PIAS-1 cDNA together with HA-Tollip wt and mutants as indicated. Immunoprecipitation with anti-HA abs; staining with anti-Flag abs. G) Western blot of protein extracts from 293T cells transfected with the Flag-PIASxβ cDNA together with the HA-Tollip wt and mutants as indicated. The protein extract was immunoprecipitated with anti-HA abs and stained with anti-Flag abs. H, I) Western blots of the same samples as in F and G respectively, stained with anti–HA abs.

The site of interaction of HA-Tollip with ARIP3 is analyzed in Fig. 2. The carboxyl- and amino-terminus deletion mutants schematized in panel A were used to identify the Tollip domain(s) interacting with ARIP3. Protein extracts from 293T cells transfected with wild type or mutant HA-Tollip and Flag-ARIP3 were immunoprecipitated with anti-HA abs and analyzed by western blot. Panel B shows the SDS-PAGE of the immunoprecipitated samples (lanes 2) next to the respective protein extracts (lanes 1) stained with anti-Flag abs. All Tollip mutants interact with ARIP3 with the exception of Δ6 where the entire N-terminus and C2 domain is removed. On the other hand, the Δ7 construct spanning aa 54–179 (C2 domain) and Δ5 spanning aa 134–274 (C-terminus of the C2 plus the CUE regions), bind ARIP3. Thus, the 134–179 peptide of the C2 region is sufficient for the interaction and contains the ARIP3 binding site. The staining with anti-HA abs (panel C) shows that all mutants are efficiently expressed (lanes 1) and immunoprecipitated (lanes 2).

We have also analyzed the Tollip interacting sites of ARIP3 (Panels D and E). ARIP3 entails 572 amino acid with three functionally characterized domains [13], [14]: 1) The SAP (SAF-A/B, Acinus PIAS) domain from aa 11–45 that may be a DNA binding site; 2) The RING domain from aa 347–388, responsible for the ligase function; 3) The androgen receptor binding site spanning aa 443–548. We have generated the three carboxyl-terminus deletion mutants shown in panel D. The wild type and mutant pCDNA3.1-Flag-ARIP3 constructs were co-transfected with pRK7-HA-Tollip cDNA. The cell extracts were immunoprecipitated with anti-HA abs and analyzed by western blot as shown in panel E. Lane 1 shows the protein extract and lane 2 the immunoprecipitated samples. The Flag-ARIP3-Δa and Δb mutants, deleted of the androgen receptor and ring domain respectively, co-immunoprecipitate with HA-Tollip while the Δc mutant, containing aa 1–169, is not detectable on the gel. This shows that HA-Tollip interacts with a region of ARIP3 spanning residues 169–347. This domain is highly conserved in the PIAS family of proteins. Thus, we investigated the possible interaction of Tollip with other members of the family. As expected, HA-Tollip interacts with both PIAS1 and PIASxβ (Fig. 2F and G). Immunoprecipitation of Flag-PIAS1 and Flag-PIASxβ with HA-Tollip deletion mutants Δ5 and Δ6 shows that the carboxyl-terminus region of the C2 domain represents the interaction site with the E3 ligases of the PIAS family that we have examined.

### Tollip triggers protein sumoylation and is itself sumoylated

**Figure 3:**
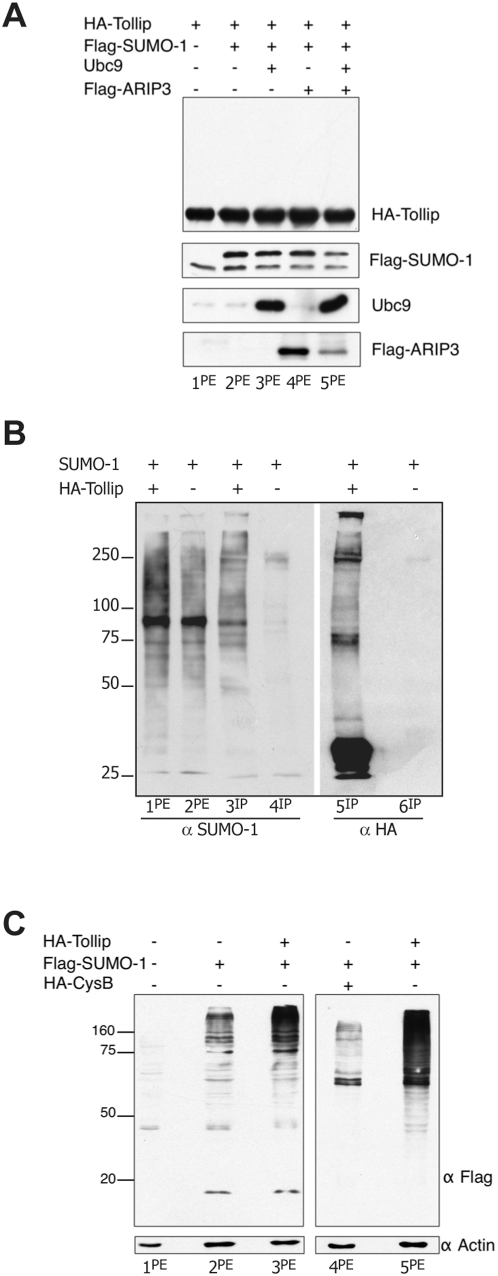
Sumoylation of Tollip. A) Western blot analysis of protein extracts from 293T cells transfected as indicated above the figure and stained for the proteins indicated on the right. B) Western blot analysis of protein extracts from 293T cells transfected with HA-Tollip and SUMO-1 (lane 1) and SUMO-1 only (lane 2) stained with anti-SUMO-1 abs. The same protein extracts were immunoprecipitated with anti-HA abs and stained with anti-SUMO-1 abs (lanes 3 and 4) and, after stripping of the membrane, they were stained with anti-HA abs (lanes 5 and 6). These samples were electrophoresed in 8% SDS-PAGE. C) Western blot of protein extracts from 293T cells transfected and stained as indicated.

The interaction of Tollip with the sumoylation enzymes suggests that Tollip is sumoylated and perhaps has a role in the sumoylation process itself. The analysis of the rat amino acid sequence (accession BC133067.1) indicates at least 3 putative sumoylation sites within the C2 domain (the lysines at position 96, 143, and 162). However, the transfection of 293T cells with HA-Tollip together with Flag-SUMO-1, Ubc9, and Flag-ARIP3 shows that, under the conditions tested, Tollip migrates as a monomer at the expected molecular weight of 29 kDa (Fig. 3A). No additional slow migrating bands can be detected. This set of experiments suggests that Tollip is not covalently modified. However, the presence of ubiquitin-like protease activity (Ulp) in native cell lysates combined with the short half-life of the SUMO-1 modification [15], [16] may destabilize the process. Thus, we have carried out an additional experiment were 293T cells were co-transfected with HA-Tollip and SUMO-1 and the cells were lysed under denaturing conditions, to block protease activity and exclude non covalent association with interacting proteins. The control cells were transfected with SUMO-1 only. The staining of the protein extracts with anti-SUMO-1 abs generates a similar pattern of bands in the two samples (Fig. 3B, lanes 1 and 2). The immunoprecipitation of the same protein extracts with anti-HA abs is shown in panel B lanes 3–6. Both SUMO-1 and anti-HA abs generate bands migrating in corresponding positions of the gel, at a molecular weight higher than monomeric Tollip (compare lanes 3 and 5). This result confirms that Tollip is sumoylated, possibly in more than one lysine residue. It should be noticed, however, that Tollip may be dimeric as well as ubiquitinated [2], [3] and this makes it difficult to relate the molecular weight to the number of SUMO-1 molecules covalently bound to it. We can conclude that in 293T cells Tollip is transiently sumoylated. The low intensity of the sumoylation bands detected in the imunoprecipitation blot may be due to the process of de-sumoylation of modified Tollip that certainly occurs. To test the possible effect of Tollip on the sumoylation process, 293T cells were transfected as indicated and the protein extracts were analyzed by western blot with anti-Flag abs (Fig. 3C). The sumoylation bands are not detectable in the protein extracts of not transfected cells (lane 1). The transfection with SUMO-1 cDNA shows a band ladder in the high molecular weight range suggesting sumoylation of numerous proteins (lane 2). Cotransfection of SUMO-1 and Tollip increases the intensity of the sumoylated bands, suggesting activation of the sumoylation process (lanes 3 and 5). This trigger is specifically due to Tollip, since overexpression of an unrelated cDNA does not activate the sumoylation process (lane 4). The intensity of the actin bands shows that the amount of proteins loaded on each lane is the same.

### The IL-1 receptor is a Tollip sumoylation target

**Figure 4:**
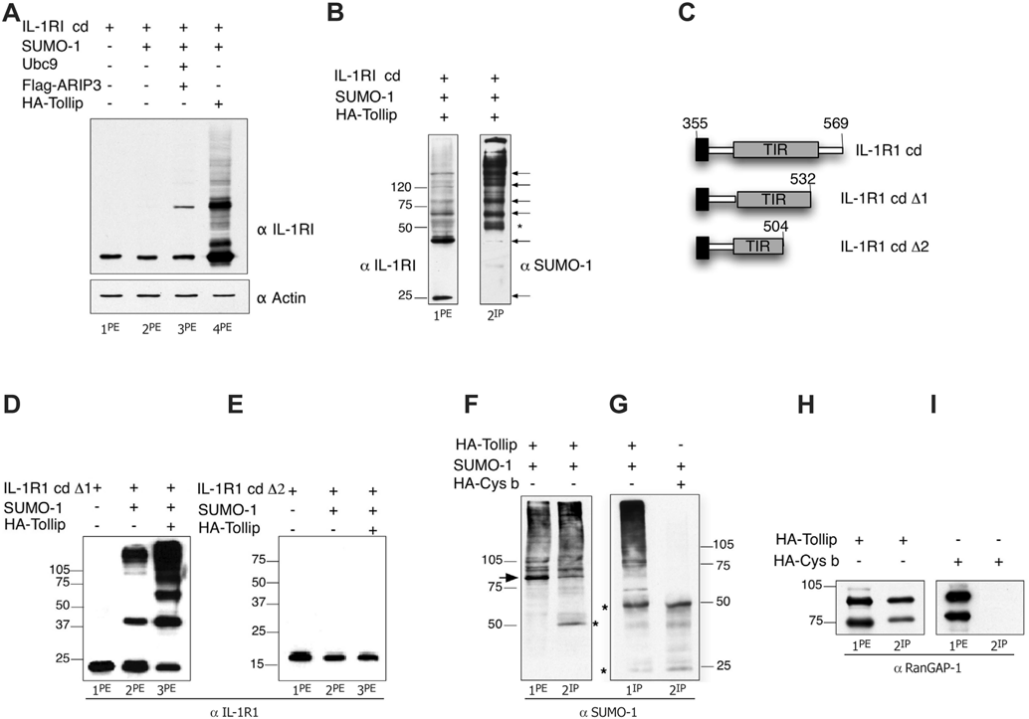
IL-1RI and RanGAP-1 are Tollip targets. A) Western blot analysis of protein extracts from 293T cells transfected as indicated above the figure. The term “cd” indicates the IL-1RI cytoplasmic domain. Staining carried out with anti-IL-1RI abs. B) Western blot analysis of protein extracts from 293T cells transfected with HA-Tollip, SUMO-1 and IL-1RI cd. Lane 1 contains the protein extract and lane 2 contains the proteins immunoprecipitated with anti-IL-1RI abs, stained as indicated. C) Schematic representation of the IL-1RI cd deletion mutants used in the experiments. The position of the TIR domain is indicated. The first and the last amminoacids present in each peptide are indicated. D) and E) Western blot analysis of protein extracts from 293T cells transfected with Δ1 and Δ2 IL-1RI mutants as indicated and stained with anti-IL-1RI abs. F) Western blot analysis of protein extract from 293T cells transfected with Flag-SUMO-1 together with HA-Tollip. The protein extract (lane 1) and the proteins immunoprecipitated with anti-HA abs (lane 2) are stained with anti-SUMO-1 abs. G) Western blot analysis of protein extracts from 293T cells transfected with Flag-SUMO-1 together with HA-Tollip (lane 1) or HA-Cystatin B (lane 2) and immunoprecipitated with anti-HA abs. H) Western blot analysis of protein extracts from 293T cells transfected as indicated above the figure, stained with anti-RanGAP-1 abs. Lanes 1 and 3 contain the protein extract and lanes 2 and 4 contain the immunoprecipitated proteins. The asterisks indicate the position of the immunoglobuline band.

The effect of Tollip on sumoylation has prompted the search for sumoylation targets. Burns et al. [1] and Brissoni et al. [8] have described the interaction of Tollip with the IL-1 receptors and its involvement with the receptor intracellular traffic. We have analyzed the possible modifications of the receptor co-transfected with the Tollip and the SUMO-1 proteins. The transfection of the full length IL-1RI receptor in 293T cells gave poor efficiency and very low expression of the protein. As the cytoplasmic region of the receptor interacts with Tollip (Fig. 1A), we have used this domain for the transfection experiments. 293T cells were transfected as indicated in Fig. 4A and the protein extracts were stained with anti IL-1RI abs. Transfection of the cytoplasmic region of the IL-1RI shows a single band migrating at about 25 KDa. Cotransfection of the cells with plasmids expressing SUMO-1 (lane 2) and the sumoylation enzymes (lane 3) only slightly modifies the banding pattern of the receptor protein. In addition to the expected band of the cytoplasmic receptor, a second band migrating at the approximate molecular weight of 40 kDa can be detected. However, the transfection of the cells with HA-Tollip together with SUMO-1 triggers the formation of a banding pattern ranging between 25 and 100 kDa molecular weight (lane 4). The western blot of the cell extract immunoprecipitated with anti-IL1RI abs and stained with anti-SUMO-1 abs shows that the band ladder contains SUMO-1 (Fig. 4B lane 2). After immunoprecipitation, at least four of the sumoylated bands stained by the abs are co-migrating with the receptor bands (compare lanes 1 and 2). This strongly suggests that sumoylation modifies the migration characteristics of the cytoplasmic domain of the receptor. We can conclude that the interaction of Tollip with the sumoylation enzymes triggers the sumoylation of IL-1RI. Figures 4D and E analyze the banding pattern of the cytoplasmic receptor in cells transfected with the Δ1 and Δ2 receptor deletion mutants indicated in C. Interestingly, the cytoplasmic mutant 355–503 (Δ2) is not sumoylated (Fig. 4E) while the fragment spanning aminoacids 355–531 (Δ1) is sumoylated (Fig. 4D). The sumoylated residues are one or more lysines present in the IL-1RI sequence 504–530 at the possible following positions: 504, 507, 509 and 527. These lysines are part of the phylogenetically conserved BOX 3 of the TIR homology region of the receptor. It should be noticed that the sumoylation of IL-1RI is stable and clearly detectable even in cells lysed in non-denaturing buffer.

A well-known sumoylation target is the GTPase activated protein RanGAP1, involved in the directional movement through the nucleo-pore complex (NPC). RanGAP1 is constitutively modified *in vivo* and remains modified even under conditions where all other SUMO-1 targets are de-sumoylated [17]. Fig. 4F shows a western blot of 293T cells transfected with HA-Tollip and Flag-SUMO-1 stained with anti-SUMO-1 abs (lane 1). The immunoprecipitation with anti-HA abs shows that Tollip interacts with sumoylated proteins and one of them migrates at the position of the sumoylated RanGAP1 (arrow) (lane 2). Fig. 4G shows the immunoprecipitation with anti-HA abs of a sample transfected as in Fig. 4F, confirming that Tollip co-immunoprecipitates with sumoylated proteins (lane1). This interaction is specific, since the immunoprecipitation of the unrelated protein HA-Cystatin B, transfected into 293T cells together with SUMO-1, does not show the same pattern (lane 2). To test whether Tollip interacts with RanGAP1 we have immunoprecipitated with anti HA abs a protein extract from 293T cells transfected with HA-Tollip or HA-cystatin B. The western blot stained with anti-RanGAP1 abs is shown in Fig. 4H. Two bands, migrating at the position of the sumoylated (approximately 90 Kd) and unsumoylated (approximately 75 Kd) RanGAP1 protein, are present in the cell extract (lane 1) and in the immunoprecipitate (lane 2). The same bands are not detectable in the control experiment (Fig. 4I, lanes 1 and 2). We conclude that Tollip interacts with both the native and sumoylated forms of RanGAP1.

### The role of sumoylated Tollip

**Figure 5:**
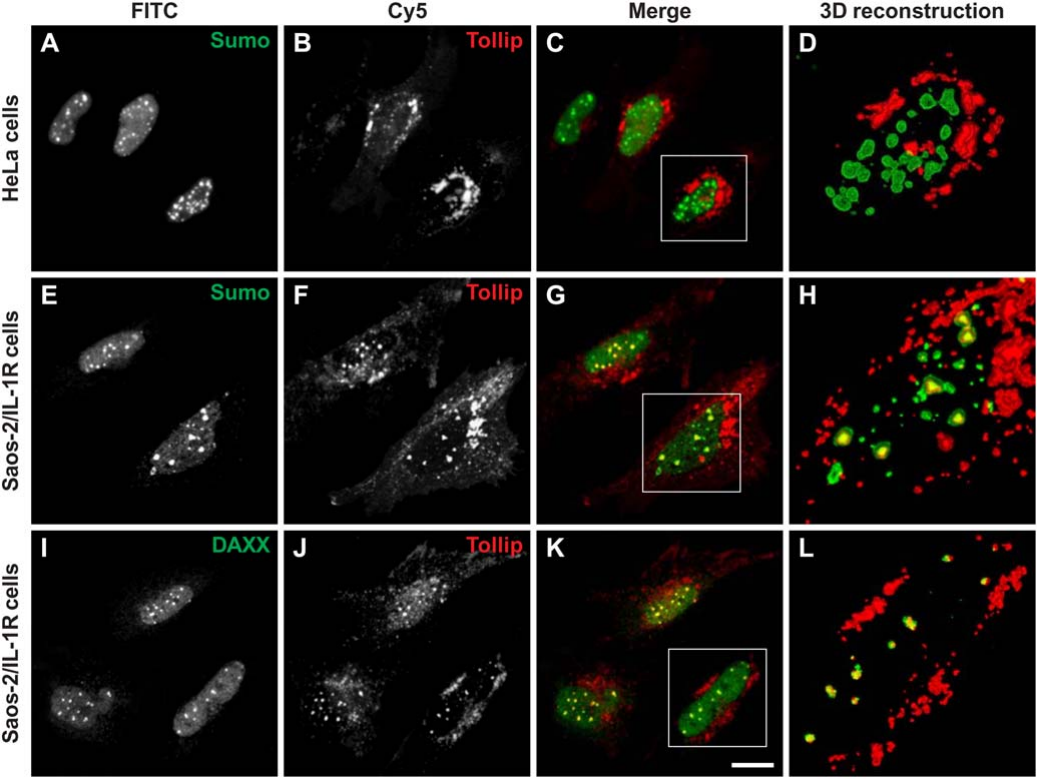
Cellular localization of Tollip. A–B) HeLa cells transiently expressing HA-Tollip and Flag-SUMO-1 stained with anti−ΗΑ (Cy5-red) and anti-Flag (FITC-green) abs. E–F) SAOS-2/IL-1RI cells transfected with HA-Tollip only and stained with anti−HA (Cy5 -red) and anti-SUMO-1 abs (FITC- green). I–J) SAOS-2/IL-1RI cells transfected with HA-Tollip only and stained with anti-HA (Cy5 -red) and anti-Daxx (FITC- green) abs. C,D,G,H,K,L) Merged reconstructed images of extended focus projections are shown. The selected areas (white rectangles) are reconstructed as three-dimensional imaging using the surface-shaded algorithm operating above a defined threshold of fluorescence intensity. The detail allows a precise evaluation of localization of the two fluorescent signals. Magnification bar: 5 µm.

So far, the function of Tollip has been linked to the endosomal degradation machinery, as an adaptor molecule, operating together with Tom1, on the ubiquitination system of protein degradation. Recently, Brissoni et al. [8] have shown that ubiquitinated Tollip is necessary to sort ubiquitinated IL-1RI at late endosomes, acting as a ubiquitin receptor. SUMO-1 is a ubiquitin-like modifier that regulates several essential cellular processes, i.e. nuclear-cytoplasmic signaling and transport, and regulation of gene expression [18]. However, sumoylation does not usually tag proteins to degradation but seems to enhance their stability, modulate their nuclear compartimentalization, sometimes competing with ubiquitin for lysine modifications, with quite a different outcome on cell function [19]. Perhaps, the most studied role of SUMO-1 modification on proteins is that of nuclear translocation [17], [9]. As we have found that Tollip can be sumoylated and interacts with Ran-GAP1, we have analyzed its cellular localization both in cells that do not express the IL-1 receptor (HeLa cells) and in cells that constitutively express IL-1RI (SAOS-2/IL1R cells). Fig. 5 panels A–D show the double fluorescence confocal analysis of HeLa cells co-transfected with SUMO-1 and Tollip. In agreement with the published data, Tollip is visible in small aggregates in the cytoplasm, in the perinuclear region in a position that may correspond to the Golgi apparatus and/or to endosomes [5]. SUMO-1 is partly diffused in the nuclear region, and partly aggregated in what may be PML bodies. None of the cells that we have examined shows colocalization between SUMO-1 and Tollip (panels C and D). In contrast, a different cellular distribution of the two proteins is observed in the human SAOS-2/IL-1R osteoblastoma cells in which IL-1RI is constitutively overexpressed. Fig. 5 panels E–H show the localization of transfected HA-Tollip and endogenous SUMO-1 in SAOS-2/IL-1R cells. The distribution of the proteins is altogether similar to that observed in HeLa cells with a major difference. The nuclear bodies in these cells show a yellow colour indicating colocalization between Tollip and SUMO-1. We conclude that, in SAOS-2/IL-1R cells, Tollip together with SUMO-1 are translocated into the nucleus and internalized into nuclear bodies. The nuclear localization of Tollip may correlate with its interaction with Daxx, which is usually found in the PML nuclear bodies. Fig. 5 panels I–L show the merging of the fluorescence of the two proteins, Tollip and Daxx that, in SAOS-2/IL-1R cells, colocalize in nuclear bodies [20]. This confirms the localization of Tollip in defined nuclear structures, and suggests a nuclear role as well as its involvement in the nuclear cytoplasmic traffic. It should be noticed that the SAOS-2/IL-1RI cells used in this experiment were transfected with HA-Tollip only, while both Daxx and SUMO-1 were the endogenous cellular components.

## Discussion

### Tollip interaction with sumoylation enzymes

This work describes for the first time the involvement of Tollip in the sumoylation process. Through the two-hybrid screening of a rat cDNA library we have identified sequences coding for the described Tollip partners: Tom1, ubiquitin and Tollip itself. However, the presence of several recombinants coding for Ubc9, Daxx, ARIP3, and one clone coding for SUMO-1 came as a novelty. These interactors strongly suggest a role of Tollip in sumoylation. The remaining clones were not further analyzed. The identification of the binding site between HA-Tollip and the Flag-tagged ligases shows that the carboxyl-terminus of the C2 domain contains the ligase binding region. In the ARIP3 ligase, the binding region is just upstream the RING domain.

A direct interaction between Tollip and the E2/E3 sumoylation enzymes suggests that the protein is sumoylated. Sumoylation is not a stable reaction and a complex network of modifying and demodifying enzymes is present both in the nucleus and in the cytoplasm and controls the modification process [21]. In fact, Tollip sumoylation is detectable only in cells lysed in 1% SDS under conditions that block protease activity. It may be noticed that the co-transfection of 293T cells with the SUMO-1 and Tollip coding plasmids is sufficient to induce Tollip sumoylation suggesting that, under these conditions, the sumoylating enzymes are not limiting. The rat Tollip sequence contains 8 lysines as potential sumoylation sites, and it is possible that the sumoylated bands immunoprecipitated with Tollip are only due to SUMO-1 modification of the protein. However, different covalent modifications and, in particular, polyubiquitylation may occur in lysine residues either competing with each other for the same site or independently regulated by events that depend on the target of interest. Although sumoylation does not usually target proteins to destruction, it has been recently shown that sumoylated proteins may become targets of E3 ubiquitylation ligases for proteosomal degradation [18], [22]. We cannot exclude that at least part of the sumoylated Tollip has a proteosomal destiny. The strong increase of the anti-SUMO-1 abs staining shown in figure 3C indicates that the overexpression of Tollip activates the overall cellular sumoylation of the proteins, thus suggesting a rather general role of Tollip in this process. Tollip sumoylation and its interaction with Ubc9 suggest a function of Tollip as a ligase. Conversely, the interaction of Tollip with three different members of the PIAS ligase family favours the idea of a sumoylation adaptor.

### Tollip mediates IL-1RI sumoylation

Our experiments identify at least one sumoylation substrate of Tollip: the IL1-RI receptor. Here we show for the first time that this receptor is sumoylated. IL-1RI sumoylation occurs within the evolutionary conserved TIR domain, which is responsible for the activation of the IL-1 signalling cascade. Furthermore, the TIR domain is the site of ubiquitylation of IL-1RI mediated by Tollip. The equilibrium between receptor ubiquitylation and sumoylation is probably important in modulating the signaling cascade of IL-1, establishing the number of receptors present on the cell surface. Tollip may play a pivotal role in modulating the IL-1RI activity through the different modifications. Hoeller et al [23] have shown that monoubiquitylated sites do not interact with polyubiquitinated proteins. It is possible that monoubiquitylated Tollip interacts with the receptor stabilizing its sumoylation. Alternatively, un-ubiquitinated Tollip interacts with the polyubiquitylated IL-1 receptor accumulating it in the late endosomes for degradation.

### Tollip has a nuclear function

In contrast with the instability of Tollip sumoylation, its expression in 293T cells seems to trigger stable modification of proteins other than Tollip, suggesting a general activation of the process following over-expression (Fig. 3C). One of the cellular proteins constitutively modified by SUMO-1 is the RanGTPase binding protein RanGAP-1, the binding partner of the RanBP2 ligase, a nucleoporine that serves as a docking site for the nuclear import of proteins [17], [9]. Our results show that transfected Tollip co-immunoprecipitates with RanGAP-1, suggesting its role in RanGAP-1 sumoylation and nuclear cytoplasmic traffic. Accordingly, for the first time we show a nuclear localization of Tollip. We do not know what the nuclear function of Tollip may be but we can speculate on some hypothesis. Nuclear Tollip may be important for the sumoylation of nuclear substrates. Alternatively, Tollip could function as a carrier for nuclear localization of sumoylated proteins, as suggested by the interaction with RanGAP-1. Tollip may be involved in the translocation and nuclear localization of the cytoplasmic portion of a number of membrane receptors, among which the sumoylated cytoplasmic IL-1RI [24]. Sumoylation regulates different cellular processes, particularly transcriptional repression and activation [19]. The regulation of transcription occurs mainly through the recruitment of transcriptional co-repressors that cause downregulation. Daxx is a transcriptional co-repressor interacting with many sumoylated factors leading to SUMO-dependent repression and Daxx interacts with Tollip. Generally, Daxx is localized in the nucleoplasm and in defined nuclear structures, i.e. in the SUMO-modified PML bodies, in the nucleolus and interacts with CENP-C in the centromere, possibly exerting different functions in the different compartments. In conclusion, here we show that Daxx interacts and colocalizes with Tollip, thus suggesting the existence of a nuclear function of Tollip, and a role of Tollip in Daxx dependent transcriptional regulation.

## Materials and Methods

### Antibodies

Anti-HA, Ubc9, SUMO-1, Daxx and IL-1RI abs were purchased from Santa Cruz. Anti-Flag and anti-Actin abs were from Sigma-Aldrich, anti-RanGAP-1 abs were from Dr. A. Dasso of the National Institute of Child Health and Development, Betsda.

### Rat cDNA Library Construction and Yeast Two hybrid Screening

Total RNA was isolated from the brain of Sprague-Dawley rats and poly(A)+ RNA was purified by affinity chromatography on oligod(T)-cellulose (Amersham Bioscence) [25]. Equal amounts of poly(A)+ RNA obtained from rat brain at 1, 2, 4, 8, 13 days of age were pooled and used to generate cDNA according to standard protocols based on the Gubler-Hoffman method [26]. The cDNA was purified using a Chroma Spin1000 column (Clontech) to select cDNAs 1 Kb long or more. The cDNA was cloned into the pJG4-5 vector obtaining 1×10^7^ independent colonies.

For the two hybrid screening, Tollip cDNA fused to the DNA-binding domain of LEX A was inserted into the pEG202 vector (bait) and transformed into the yeast strain W303 (MATa) together with the LacZ reporter plasmid pSH18-34. The cDNA library was used to transform the EGY48 (matα) yeast strain to generate a pre-transformed yeast library. The bait strain containing pEG202/Tollip was crossed with an aliquot of the pre-transformed library strain and 5×10^7^ diploids were screened for interactors [27]. Positive clones were confirmed in a second round of screening in yeast and sequenced.

### Ethical Issues

Experiments were authorized by the local bioethical Committee and performed according to the Italian and European Community legislation on the use of animals for experimental purposes.

### In vitro transcription, translation, and GST pull-down assays

The Tollip cDNA sequence was inserted into the pGEX4T1 vector (Amersham Bioscence) in fusion with the Glutathione S-Transferase (GST) protein. The GST-Tollip fusion protein was expressed in *E. coli* cells and purified by binding to Glutathione-Sepharose beads. In vitro transcription of the identified interactors was carried out using the Riboprobe in vitro Transcription System (Promega) according to the manufacturer instructions. In vitro translation was carried out using the Rabbit Reticulocyte Lysate System (Promega) in presence of ^35^S-methionine (Amersham Bioscience). The GST pull-down assays were carried out by incubating equal amounts of GST or GST-Tollip immobilised to Gluthathione-Sepharose beads (Amersham Bioscence) with 5 µl of ^35^S-labelled translation product in 100 µl final volume (binding buffer: 20 mM sodium phosphate pH 6, 150 mM NaCl, 10% glycerol, 1 mM PMSF, 1 mM DTT, 0.02% Nonidet P-40) The mixtures were incubated 3 h at room temperature and washed three times with binding buffer. Bound proteins were eluted with 2× SDS buffer, separated by SDS-PAGE and visualised by autoradiography.

### Cell lines and cDNA constructs

293T and SAOS-2/IL-1RI (overexpressing IL-1RI) cells were grown in Dulbecco's Modified Eagle Medium (DMEM) supplemented with 10% fetal bovine serum (FBS) at 37°C and 5% CO_2_. The cDNAs coding for rat Tollip wild type and its deletion mutants were cloned into the pRK7-HA expression vector. The cDNAs coding for rat Ubc9, the cytoplasmic region of the rat IL-1RI receptor and its mutants were cloned into the pRK7 expression vector. The cytoplasmic portion of IL-1RI spans aminoacid 355–569 of the rat cDNA sequence (accession NP_037255). The cDNA coding for rat SUMO-1, PIAS-1, PIASxb, ARIP3 and its deletion mutants were cloned into the pcDNA3.1-Flag-C expression vector, which is a modified version of the pcDNA3.1-His C plasmid (Clontech) where the hystidine tag sequence was replaced with a Flag coding sequence. The cDNA recombinant clones were amplified by PCR from the rat cDNA library used for the two hybrid screening. Transfection of 293T cells with the expression vectors was performed using Polyethylenimine (PEI) (Sigma Aldrich) according to manufacturer instructions.

### Immunoprecipitation and western blot analysis

293T cells were lyzed, 24 hr after transfection, in RIPA buffer (300 mM NaCl, 0.5% NP40, 20 mM Hepes KOH pH 8, 20% Glycerol, 2 mM DTT, 0.4 mM EDTA pH 8) and sonicated. Lysis under denaturing conditions was carried in 1× PBS containing 1% SDS. The samples were boiled 10 min and diluted 10 times in PBS, containing a cocktail of anti-proteases and anti-phosphatases (Sigma), to a final concentration of 0.1% SDS. The diluted samples were sonicated. 400 µg protein extracts were immunoprecipitated overnight with the specific abs and separated on Protein A-Sepharose beads (Amersham Biosciences). 40 µg protein extracts were loaded on the gel to check the efficiency of the transfection.

Unless otherwise stated, the protein extracts and immunoprecipitates were analyzed in 12% SDS-PAGE. The western blots were carried out using the BioRad apparatus according to the manufacturer instructions. Staining was carried out with the ECL Western Blot Detection Reagent (Amersham Biosciences) according to the instructions.

### Immunofluorescence

SAOS-2/IL-1RI and HeLa cells were seeded onto collagen-coated glass covers and, after 24 hr, fixed in 4% PFA for 20′ at 4°C. Following 5′ incubation in 0.1% Triton X-20, 3%BSA the cells were stained with mouse anti-Tollip and rabbit anti-SUMO-1 abs followed by incubation with FITC conjugated anti-mouse and Cy3 conjugated anti-rabbit secondary abs. After staining, the cells were analyzed by confocal microscopy. Confocal images were acquired using a Radiance 2000 confocal laser scanning microscope (BioRad), equipped with a Nikon x60, oil immersion 1.4 N.A. objective and with krypton and red diode lasers. For FITC and Cy5 double detection, the samples were simultaneously excited with the 488-nm line of the krypton laser and with the 637-nm line of the red diode laser. The emission signals from FITC and Cy5 were separated by a dichroic mirror (DM; 560 nm) and simultaneously detected by two photomultiplier tubes. Two barrier filters (BP; 515/30 nm for FITC and LP; 660 nm for Cy5) were placed before the two photomultiplier tubes to minimize the overlap between the two signals, as previously described. Optical sections were obtained at increments of 0.3 µm in the Z-axis and were digitized with a scanning mode format of 512×512 pixels and 256 grey levels. Image processing and volume rendering (extended focus and surface-shaded) were carried out using the ImageSpace software (Molecular Dynamics, Sunnyvale, CA) running on a workstation Indigo (Silicon Graphics, Mountain View, CA.).
